# On a Case of Poisoning from Swallowing Chloroform, and on Its Administration in Lead Colic

**Published:** 1852-12

**Authors:** 


*On a Case of Poisoning from swallowing Chloroform, and on its Administration in Lead Colic.* By Dr. Aran.—This occurred in a case of severe lead colic, for which the chloroform was given internally with advantage, when on the eighth day of treatment, the patient by mistake drank a draught from the bottle containing the chloroform. The burning sensation produced informing him of his error, he drank large quantities of water, and made ineffectual attempts to vomit. He was found a few minutes afterwards with his eyes shining, his features animated, singing and talking incoherently, and unable to recognize those about him. There were various convulsive movements, and the skin was insensible to pinching, pricking, &c. The pupils acted naturally, but the power of vision seemed gone. Pulse between seventy and eighty. In twenty or thirty minutes he fell into a sleep, which became very deep, and was accompanied by anæsthesia of surface and complete relaxation of limbs, the pupils and respiration continuing normal. Leeches were applied behind the ears, and purgative enemata given, and in a few hours he rose like a drunken man, to fall into a sound sleep again. Next day, he could remember nothing of what occurred, and the ill effects gradually wore off. As near as could be guessed, the man must have drank between eight and ten drachms of chloroform; and the innocuity of so large a dose can only be explained by the rapid elimination which Snow and others have proved that chloroform undergoes. M. Aran thinks that in similar cases, a preferable treatment would be the administration of strong coffee, and the application of cold to the head and sinapisms to the feet—means whose efficacy is known in cases of poisoning by alcohol, opium, ether, &c.

M. Aran speaks in warm terms of the internal use of chloroform in hysteria, spasmodic colic, lead colic, &c., given in increasing doses from 20 to 150 drops in the twenty-four hours. Not only has no accident occurred to any of the very numerous cases in which he has given it, but the physiological effects have at most in some cases resembled the transient intoxication induced by champagne. He has compared its efficacy in *lead colic* with that of the usual remedies, especially purgatives and alum, in a great number of cases. He does not deny the efficacy of *purgatives*, and thinks that they should be always employed in conjunction; but when given alone, only very powerful ones are efficacious, and then only temporarily so. He thinks even less favorably of *alum*, as he has found it very slow in operation, and of doubtful efficacy, beyond enabling mild aperients to act where drastics would otherwise have been required. *Chloroform* acts as opium or belladonna, by relieving the spasm, which constitutes so important an element in the disease. It has this advantage, that it may be given in considerable doses, without the incessant watching required by the other two, owing to its rapid elimination. It should not, any more than they, be employed alone, but in conjunction with means for ridding the economy of the lead, as sulphur and vapour baths, &c. The chloroform is to be applied locally, and administered in a mixture, in doses of from fifteen to twenty drops morning and evening, and as much in a glyster, increasing this quantity if required.—*Bull. de Therap.*—*Ibid.*

